# An Innovative Mental Health Model for Treating Culturally Diverse Older Adults

**DOI:** 10.1093/geroni/igaa057.306

**Published:** 2020-12-16

**Authors:** Tobi Abramson, Jacquelin Berman, Madison Gates

**Affiliations:** 1 NYC Department for the Aging, NYC, New York, United States; 2 NYC Department for the Aging, New York city, New York, United States

## Abstract

The mental health needs of older adults are largely unmet, a finding even more prevalent within culturally diverse older adult populations. Added to this is the high rate of social isolation. Research has indicated increased connection to mental health services when services are embedded within physical health care settings. For those attending community centers, 85% indicate that they are socially isolated, 68% indicate they are lonely, and 53% have a mental health need (compared to 20% nationally). The need for innovative programming is evident. When examining the needs of diverse older adults, it is increasingly important that new and innovative approaches address social isolation, loneliness, and mental health problems experienced by this cohort. Utilizing this knowledge an innovative model of embedding and integrating mental health services, provided by bilingual and bicultural clinicians, into congregate sites (older adult centers) was implemented. Those that participated were mainly female (72.1%), 68.5% English-speaking, 14.5% Spanish-speaking, 13.6% Chinese-speaking and 3.4% other. Spanish-speakers had more depression than English-speakers and both had more depression than Chinese-speakers. English and Spanish-speakers reported more social isolation and Chinese-speakers compared were more likely to participate in engagement. Chinese-speakers were less likely to be in clinical services with a positive screen compared to English-speakers. Overall, 75% engaged in treatment; 37.3% and 41% showed a 3-month improvement of depression and anxiety, respectively. This presentation focuses on the innovative components of this model, how to engage diverse older adults to utilize treatment, steps needed for replication, and policy implications around integrated mental health treatment.

